# BAFF attenuates oxidative stress-induced cell death by the regulation of mitochondria membrane potential via Syk activation in WiL2-NS B lymphoblasts

**DOI:** 10.1038/s41598-020-68628-5

**Published:** 2020-07-16

**Authors:** Sojin Park, Ju-Won Jang, Eun-Yi Moon

**Affiliations:** Department of Integrative Bioscience and Biotechnology, Sejong University, 209 Neungdong-ro Kwangjin-gu, Seoul, 05006 Republic of Korea

**Keywords:** Mechanisms of disease, Target validation

## Abstract

Cell survival is facilitated by the maintenance of mitochondrial membrane potential (MMP). B cell activating factor (BAFF) plays a role in survival, differentiation, and maturation of B cells. In the present study, we examined whether BAFF could attenuate oxidative stress-induced B cell death by the regulation of MMP collapse via spleen tyrosine kinase (Syk) activation using WiL2-NS human B lymphoblast cells. BAFF binds to receptors on WiL2-NS cells. When the cells were incubated in serum-deprived conditions with 1% fetal bovine serum (FBS), BAFF reduced the percentage of dead cells as determined through trypan blue staining and caspase 3 activity. BAFF also inhibited MMP collapse with 1% FBS, as indicated by a decrease in the number of cells with high-red fluorescence of MitoProbe™ JC-1 reagent or a decrease in the percentage of DiOC_6_-stained cells. Reactive oxygen species (ROS) production was reduced by incubation with BAFF in the presence of 10% or 1% FBS. BAFF inhibited MMP collapse, cell growth retardation, dead cell formation, and caspase 3 activation caused by treatment with H_2_O_2_. Syk phosphorylation on tyrosine (Y) 525/526 was increased in cells incubated with 1% FBS in the presence of BAFF than cells incubated with 1% FBS or BAFF alone. BAY61-3606, a Syk inhibitor reduced the effect of BAFF on MMP collapse, caspase 3 activation, cell growth retardation, and dead cell formation. Together, these data demonstrate that BAFF might attenuate oxidative stress-induced B cell death and growth retardation by the maintenance of MMP through Syk activation by Y525/526 phosphorylation. Therefore, BAFF and Syk might be therapeutic targets in the pathogenesis of B cell-associated diseases such as autoimmune disease.

## Introduction

B cell activating factor (BAFF), also known as TALL-1, BLyS, THANK, and TNFSF13B, belongs to the tumor necrosis factor (TNF) superfamily^1^. BAFF is expressed in both immune cells such as dendritic cells, macrophages, and T cells^1^ and non-immune cells such as alveolar-associated cells^2^, fibroblast-like synoviocytes^3^, and various epithelial cells^4^. BAFF naturally exists as a transmembrane protein and is solubilized by proteolytic cleavage of furin convertase between R133 and A134^5,6^. BAFF transmits signals by binding to BAFF receptors, BAFF-R, transmembrane activator and calcium-modulator and cytophilin ligand interactor (TACI), and B cell maturation antigen (BCMA), with different binding affinities. Weak BAFF stimuli cause a deficiency of mature B cells^7,8^. In contrast, excessive systemic humoral immune responses by elevated BAFF levels lead to the accumulation of antigen–antibody immune complexes, which in turn lead to inflammatory responses and aggravate autoimmune diseases such as rheumatoid arthritis (RA), systemic lupus erythematosus (SLE), and type 1 diabetes^1,9,10^.

Inflammation is a general symptom of autoimmune disease and induces large quantities of reactive oxygen species (ROS) in disease sites^11–13^. Highly reactive unpaired electrons in ROS can cause tissue damage, leading to apoptosis or necrosis^14^. In the process of cell death, the electron transport chain in the mitochondria is disrupted^15^, resulting in the collapse of the mitochondria membrane potential (MMP), and the hydrogen ion concentration gradient between the mitochondrial intermembrane space and the matrix^16^. The maintenance of MMP is crucial for ATP production^17^ and cell survival^15^. Therefore, the collapse of MMP is regarded as a pro-apoptosis signal^18^. BAFF also reportedly up-regulates MMP through protein kinase B (AKT) and PKC-β in mouse B cells^19^. Meanwhile, another study found that BAFF expression was increased by ROS in mouse macrophages^20^. However, little is known about the effects of BAFF on ROS production in human B cells.

Spleen tyrosine kinase (Syk) is a major signal transmitter that contributes to B cell survival and differentiation^21,22^. Syk consists of many tyrosine (Y) residues that can be phosphorylated, and the phosphorylation of Y323, Y352, and Y525/526 leads to various pathways^9,23–25^. Among the phosphorylation sites in Syk^26^, Y525/526 is in a kinase domain that is important for kinase activity^27^. Syk is activated by ROS^28–30^ in chicken B cells and BAFF in mouse B cells^31^. However, little is known about the effects of BAFF on MMP by ROS production via Syk in human B cells.

The role of Syk on cell fate varies depending on the cell type and cellular conditions. Oxidative stress-induced Syk activation activates different pathways, such as pro-apoptotic and survival pathways. Therefore, a balance of pro-apoptotic and survival pathways are important for determining the fate of cells exposed to oxidative stress^13^. Syk expression protects cells from apoptosis induced by oxidative or genotoxic stress in MCF7 and MDA-MB-231 breast cancer cells and DG75 B-lymphoma cells^32^. Syk-deficient B cells can survive in the periphery for an extended period of time^33^. Syk inhibition is a promising therapeutic strategy to disrupt pro-survival microenvironment signaling in chronic lymphocytic leukemia^34^. Syk is also involved in the induction of G2/M arrest, which protects cells from apoptosis. Syk-dependent PLC-gamma2 activation was required for acceleration towards apoptosis following oxidative stress^13^. These reports from different groups yielded contradictory results, so it is necessary to clarify the connection between BAFF signaling and Syk phosphorylation in B cells.

In this study, we investigated whether BAFF could ameliorate B cell survival through the inhibition of ROS production, MMP collapse, and Syk activation. To do this, we incubated WiL2-NS human B lymphoblast cells isolated from the spleen of a Caucasian male (American Type Culture Collection) in serum-deprived conditions (1% FBS). Our results suggest that Syk is a key regulator for the maintenance of MMP by BAFF in human B cells.

## Materials and methods

### Reagents

Recombinant human BAFF, biotinylated anti-human BAFF-R and TACI antibodies was purchased from R&D Systems (Minneapolis, MN, USA). Human BAFF-murine CD8 (BAFF-muCD8), biotinylated fusion proteins, were purchased from Ancell (Bayport, MN, USA). BAY61-3606, Syk inhibitor, was purchased from Adooq bioscience (Irvine, CA, USA). Catalase, dimethyl sulfoxide (DMSO), *N*-acetyl-L-cysteine (NAC), hydrogen peroxide (H_2_O_2_) and 3,3′-dihexyloxacarbocyanine iodide (DiOC_6_) were purchased from Sigma-Aldrich (St. Louis, MO, USA). 5,5′,6,6′-tetrachloro-1,1′,3,3′-tetraethylbenzimidazolocarbocyanine iodide (JC-1) was obtained from Invitrogen (Eugene, Oregon, USA). 2′,7′–dichlorofluorescin diacetate (DCF-DA) was purchased from Molecular Probe (Eugene, Oregon, USA). Ac-DEVD-pNA, caspase 3 substrate, was obtained from Santa Cruz Biotechnology (Santa cruz, CA, USA). Antibodies to Syk and phospho-Syk (Y525/526) were purchased from Cell Signaling Technology (Berverly, MA, USA). Except indicated, all chemicals were obtained from Sigma-Aldrich (St. Louis, MO, USA).

### Cell cultures

WiL2-NS (ATCC® CRL-8155™), a human B lymphoblast cells were provided from the Korea Research Institute of Bioscience and Biotechnology (KRIBB) cell bank (Daejeon, Korea). Cells were incubated in RPMI 1640 medium (GIBCO, Grand Island, NY, USA) with 10% heat-inactivated fetal bovine serum (FBS) (GIBCO, Grand Island, NY, USA), 2 mM L-glutamine and 100 units/ml of penicillin/streptomycin (GIBCO, Grand Island, NY, USA) at 37 °C humidified incubator under the 5% CO_2_ condition^35^.

### Measurement of BAFF-BAFF receptor interactions

BAFF binding on BAFF receptors were determined by the method previously described^36^. Briefly, WIL2-NS cells were suspended in 100 μl of Hank’s Balanced Salt Solution (HBSS) complemented with 2% bovine calf serum (BCS). Then, cells were incubated with 10 μg/ml of a human BAFF-murine CD8 (BAFF-muCD8) biotinylated fusion protein on ice for 30 min. After washing cells with 2% BCS-containing HBSS twice, cells were incubated with phycoerythrin (PE)-conjugated streptavidin (BD pharmingen, SanJose, CA, USA) for 20 min on ice. After washing cells with 2% BCS-containing HBSS twice, fluorescence intensity of 10,000 cells was analyzed by FACSCalibur™ (Becton Dickinson, San Joes, CA, USA).

### Measurement of reactive oxygen species

Intracellular ROS level was measured by incubating cells with or without 10 μM DCF-DA at 37 °C for 30 min. Flourescence intensity of 10,000 cells was analyzed by FACSCalibur™ (Becton Dickinson, San Joes, CA, USA)^35^.

### Measurement of mitochondrial membrane potential

To measure mitochondrial membrane potential (MMP), cells were stained with 2.5 μg/ml JC-1 or DiOC_6_ for 10 min at 37 °C. Then, cells stained with JC-1 were observed by 485 nm filter of fluorescence microscope and five areas were pictured to count number of high red fluorescent (J-aggregate) live cells. Dead or dying cells exhibit yellow or green fluorescence (J-monomer) with collapsed mitochondrial potential, respectively. Data were represented by the percentage of high red-fluorescence cells^37^. In addition, cells stained with DiOC_6_ were analyzed by FACSCalibur™ (Becton Dickinson, San Joes, CA, USA). Cells with MMP collapse showed a decrease in DiOC_6_ fluorescence intensity.

### Measurement of caspase 3 activity

According to previous report^37^, 1 × 10^7 ^cells were harvested and lysed in 100 μl lysis buffer containing 50 mM 2-[4-(2-hydroxyethyl)piperazin-1-yl]ethanesulfonic acid (HEPES, pH 7.4), 5 mM (3-[(3-cholamidopropyl)dimethylammonio]-1-propanesulfonate (CHAPS), and 5 mM dithiothreitol (DTT). for 20 min on ice. Then, cell lysates were centrifuged by 16,000 × *g* for 10 min at 4 °C. After centrifugation, supernatants were transferred into new tube. Each 5 μl supernatant sample was incubated with 200 μM of caspase 3 substrate (Ac-DEVD-pNA) in assay buffer containing 20 mM HEPES (pH 7.4), 0.1% CHAPS, 5 mM DTT, and 2 mM ethylenediaminetetraacetic acid (EDTA). Total incubation volume was 100 μl per each well in 96 well plate, After incubation for 2 h, optical density was measured by ELISA reader (Molecular Devices, Sunnyvale, CA) at 405 nm. Each sample was normalized by protein concentration.

### Trypan blue exclusion assay

Diluted cell suspension was mixed with equal volume of 0.4% trypan blue in PBS. Dying or dead cells were stained with blue color and viable cells were unstained. Each cell was counted by using hemocytometer under light microscope (Olympus Korea Co., Ltd, Seoul, Rep. of Korea)^35^.

### Western blot analysis

As reported previously^35^, cellular proteins were extracted by 0.5% Nonidet P-40 lysis buffer containing 20 mM Tris-HCl (pH 8.2), 150 mM NaCl, protease inhibitor (2 μg/ml aprotinin, 2 μg/ml pepstatin, 1 μg/ml leupeptin, 1 mM phenylmethylsulfonyl fluoride) and phosphatase inhibitor (1 mM sodium vanadate and 5 mM sodium fluoride). Cells were lysed for 30 min on ice and centrifuged at 13,000 rpm for 20 min at 4 °C. Protein concentrations of lysates were determined by using SMART™ BCA protein assay kit (iNtRON, Gyeonggido, Korea). Equal amounts of cellular proteins in sodium dodecyl sulfate (SDS) sample buffer were denatured by boiling at 100 °C for 5 min. Samples were separated according to protein size by SDS-PAGE (sodium dodecyl sulfate–polyacrylamide gel electrophoresis). Separated samples were transferred to nitrocellulose membrane. Membranes were blocked with 2% skim milk in Tris buffered saline containing 0.5% Tween20. After blocking, protein expression of each sample was probed by immune-reaction using enhanced chemiluminescence.

### Statistical analyses

Experimental differences were examined separately for statistical significance using ANOVA and Students’ t-distribution. The *p* value of < 0.05 or < 0.01 was considered to be significant^35,37^.

## Results

### BAFF rescued cells from oxidative stress-induced cell death

**Figure 1:**
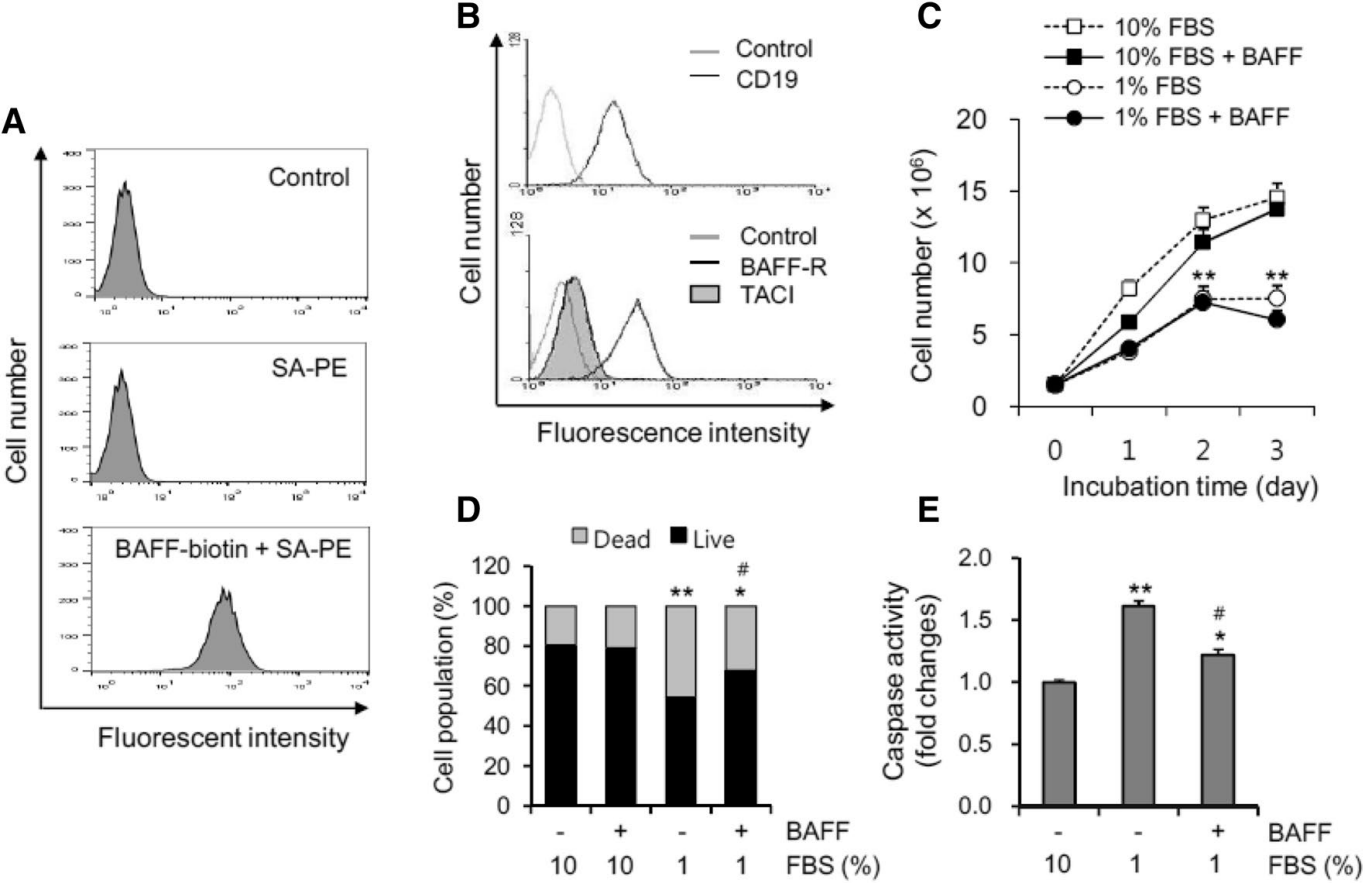
Oxidative stress-induced cell death was inhibited by BAFF. (**A**) WiL2-NS cells were incubated with 10 μg/ml of a human BAFF-murine CD8 (BAFF-muCD8) biotinylated fusion protein on ice for 30 min. BAFF binding was visualized the incubation with phycoerythrin (PE)-conjugated streptavidin (SA) for 20 min and analyzed by flow cytometry. (**B**) WiL2-NS cells were incubated with biotinylated anti-human BAFF-R or TACI antibodies for 30 min followed by PE-conjugated SA for 20 min. At the same time, cells were incubated with FITC-labeled CD19. Expression of BAFF-R or TACI and CD19 on each cell was analyzed by flow cytometry as compared to control group incubated with isotype antibodies. (**C**) and (**D**) Cells were incubated in the RPMI 1640 medium with 10% or 1% FBS in the presence or absence of 20 ng/ml BAFF. Total number of cells (**C**) or dead cells (**D**) were counted with hemocytometer and estimated by trypan blue staining, respectively. Cell lysates were prepared and caspase 3 activity was measured by using Ac-DEVD-pNA, caspase 3 substrate. Caspase 3 activity was normalized with protein concentration (**E**). Data were the representative of four experiments. Data in the line (**C**) or bar (**D–E**) graph represent the means ± SD. *p < 0.05; **p < 0.01; significant difference as compared to BAFF-untreated control with 10% FBS (**C, D** and **E**). ^#^*p* < 0.05; significant difference as compared to BAFF-untreated control with 1% FBS (**D** and **E**).

To examine the effects of BAFF on a human B cell line, we determined whether BAFF could bind to the cell surface of WiL2-NS human B cells that expressed BAFF receptors. To do this, cells were incubated with biotin-labelled BAFF or antibodies to BAFF receptors. The results revealed a significant increase in BAFF binding on the surface of WiL2-NS cells (Fig. 1A). The expression level of BAFF-R was higher than that of TACI (Fig. 1B). However, no BCMA expression was detected (data not shown), which suggests that BAFF might affect WiL2-NS cells via BAFF binding on the cell surface. Next, we used WiL2-NS cells to assess the effect of BAFF on molecular and cellular changes that control B cell death or survival.

BAFF increases B cell survival^1,9,10^ and is associated with an increase in the incidence of autoimmune disease. Additionally, serum deprivation (SD) leads to apoptotic cell death^38,39^; therefore, we examined the effects of BAFF on oxidative stress-induced cell death by using 1% rather than 10% FBS. As shown in Fig. 1C, the cell number was reduced to about half by incubation with 1% FBS compared to 10% FBS. When cells were incubated with 10% or 1% FBS-containing medium in the presence or absence of BAFF, no changes were detected in total cell number. The number of dead cells was determined using a trypan blue staining assay. The percentage of cell survival with 1% FBS decreased to about 50% as compared to about 80% with 10% FBS. When cells were incubated with 1% FBS-containing medium in the presence of BAFF, the percentage of cell survival was rescued to about 70% (Fig. 1D). In 1% FBS, a caspase 3 assay revealed that cell death increased by about 1.6 times compared to that with 10% FBS. Caspase 3 activity induced by 1% FBS decreased about 1.2 times by treatment with BAFF (Fig. 1E). This suggests that BAFF binding might activate or change molecules or intracellular events to control B cell death.

### BAFF attenuated MMP collapse in serum-deprived (SD) conditions

**Figure 2:**
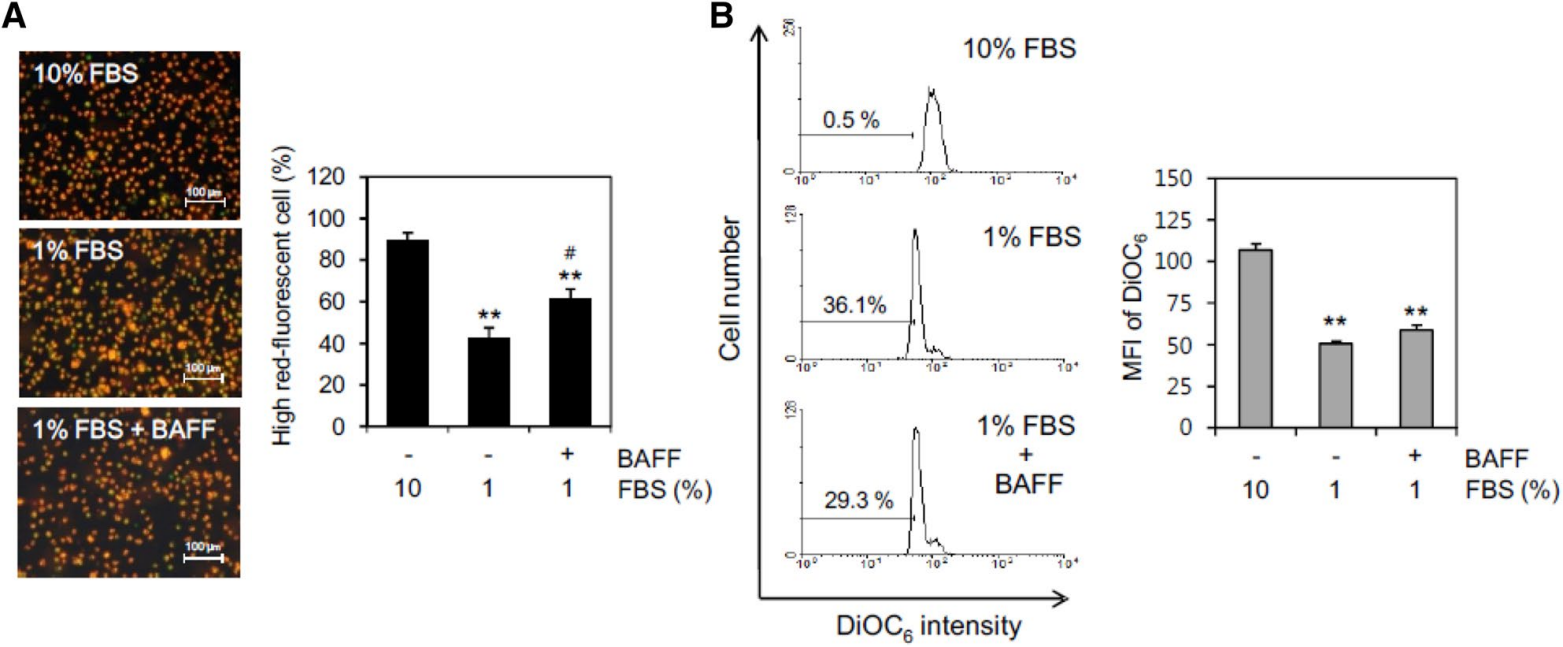
BAFF attenuated MMP collapse by the incubation with 1% FBS. (**A**, **B**) WiL2-NS cells were incubated in the RPMI 1640 medium with 10% or 1% FBS in the presence or absence of 20 ng/ml BAFF. Then, cells were stained with MitoProbe™ JC-1 reagent (**A**) or DiOC_6_ (**B**) for the detection of MMP. Cells were observed and photographed with 400 × magnification under fluorescence microscope (**A**, left). Number of high-red fluorescent cells was counted and represented as bar graph with mean ± SD (**A**, right). Percentage of cells with low fluorescence of DiOC_6_ was analyzed by flow cytometry. Mean fluorescence intensity (MFI) in each histogram was analyzed with software FlowJo V10 (**B,** left). MFI was represented as bar graph with the means ± SD (**B,** right). All experiments were performed four times. **p < 0.01; significant difference as compared to BAFF-untreated control with 10% FBS. ^#^*p* < 0.05; significant difference as compared to BAFF-untreated control with 1% FBS.

MMP plays a role in the regulation of various cellular functions^37,40^, and SD induces apoptotic cell death of rat retinal ganglion cells via mitochondrial changes^38^. Therefore, we determined the effects of BAFF on MMP changes using MitoProbe™ JC-1 reagent in WiL2-NS cells that were incubated with 1% FBS. The number of live cells with high red fluorescence was determined to assess the MMP of cells incubated with 1% FBS-containing medium. The MMP decreased to about 40% as compared to 83% with 10% FBS. When cells were incubated with 1% FBS-containing medium in the presence of BAFF, the MMP was significantly rescued to about 60% (Fig. 2A). MMP changes by 1% FBS were also determined by incubation with DiOC_6_. As shown in Fig. 2B, the fluorescence intensity of DiOC_6_ was decreased by incubation with 1% FBS. The percentage of cells with low fluorescence intensity of DiOC_6_ increased to about 36% as compared to 0.5% with 10% FBS. The mean fluorescence intensity (MFI) of the cell population decreased to about 50.5 as compared to 106.8 with 10% FBS. When cells were incubated with 1% FBS-containing medium in the presence of BAFF, the percentage of cells with low fluorescence intensity of DiOC_6_ decreased to about 29% and the MFI of the cell population increased to about 58.6. This suggests that BAFF could regulate B cell survival by controlling MMP via BAFF-mediated activation of molecules.

### BAFF reduced ROS production in serum-deprived (SD) conditions

**Figure 3:**
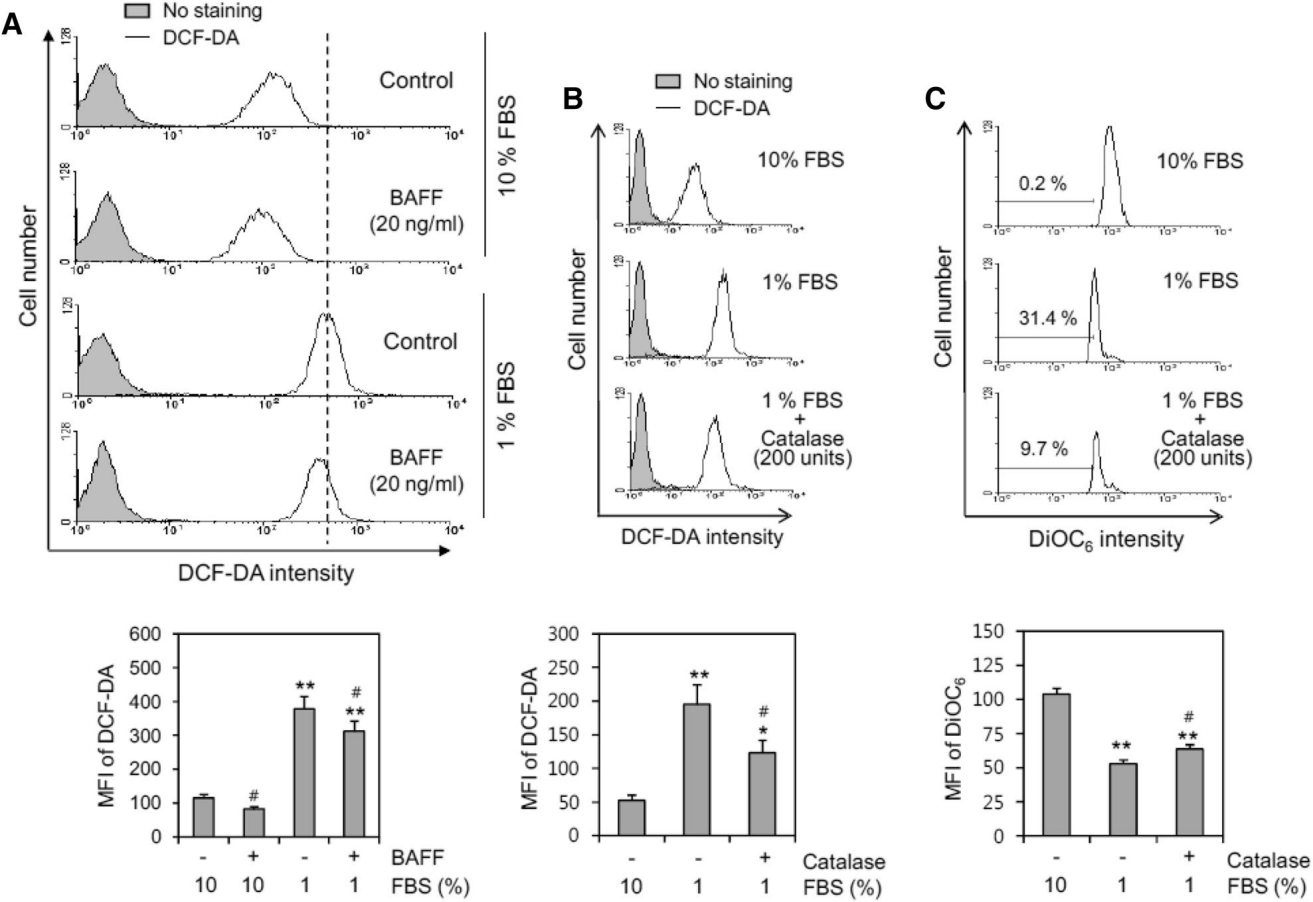
BAFF inhibited ROS level increased by the incubation with 1% FBS. (**A–C**) WiL2-NS cells were incubated in the RPMI 1640 medium with 10% or 1% FBS in the presence or absence of 20 ng/ml BAFF (**A**) or 200 units catalase (**B**, **C**) for 12 h. Then, cells were incubated with DCF-DA and analyzed by flow cytometry (**A**, **B**). Mean fluorescence intensity (MFI) in each histogram was analyzed with software FlowJo V10 (**A–C**). Cells were stained with DiOC_6_ for the detection of MMP. Percentage of cells with low fluorescence of DiOC_6_ was analyzed by flow cytometry (**C**). All experiments were performed four times. Mean fluorescence intensity (MFI) was represented as bar graph with the means ± SD (**A–C**, bottom). *p < 0.05; **p < 0.01; significant difference as compared to BAFF-untreated control with 10% FBS. ^#^*p* < 0.05; significant difference as compared to BAFF-untreated control with 1% FBS.

Because ROS are reportedly the main cause of MMP collapse in various cells^16,37,38,40,41^, we examined the changes in intracellular ROS levels by incubation with 1% FBS using dihydrodichlorofluorescein diacetate (DCF-DA). As shown in Fig. 3A, the ROS level was significantly increased by incubation with 1% FBS as compared with 10% FBS. The MFI of the cell population in 10% and 1% FBS was 114 and 377, respectively. When cells were incubated with 1% FBS-containing medium in the presence of BAFF, the ROS level significantly decreased, as judged by a MFI of 311. This suggests that BAFF could regulate B cell survival by controlling MMP via BAFF binding-mediated regulation of ROS production. The increase in ROS level in the presence of 1% FBS was reduced by treatment with catalase, which catalyzes the decomposition of hydrogen peroxide (H_2_O_2_) to water and oxygen. The MFI of the cell population in 1% FBS was 195.5, which was reduced to 123.1 by incubation with catalase as compared to 53.2 in the control group in 10% FBS (Fig. 3B). As shown in Fig. 3C, catalase also reduced the extent of MMP collapse, which was determined by incubation with DiOC_6_. The MFI of the cell population in 1% FBS was 52.9, which increased to 63.7 by incubation with catalase as compared to 103.8 in the control group (10% FBS). In addition, the percentage of cells with  low fluorescence of DiOC_6_ in 1% FBS was 31.4%, which decreased to 9.7% as compared to 0.2% in the control group. This suggests that cell death might be mediated by ROS, which can cause H_2_O_2_-induced MMP collapse.

### BAFF inhibited H_2_O_2_-induced MMP collapse and B cell death

**Figure 4:**
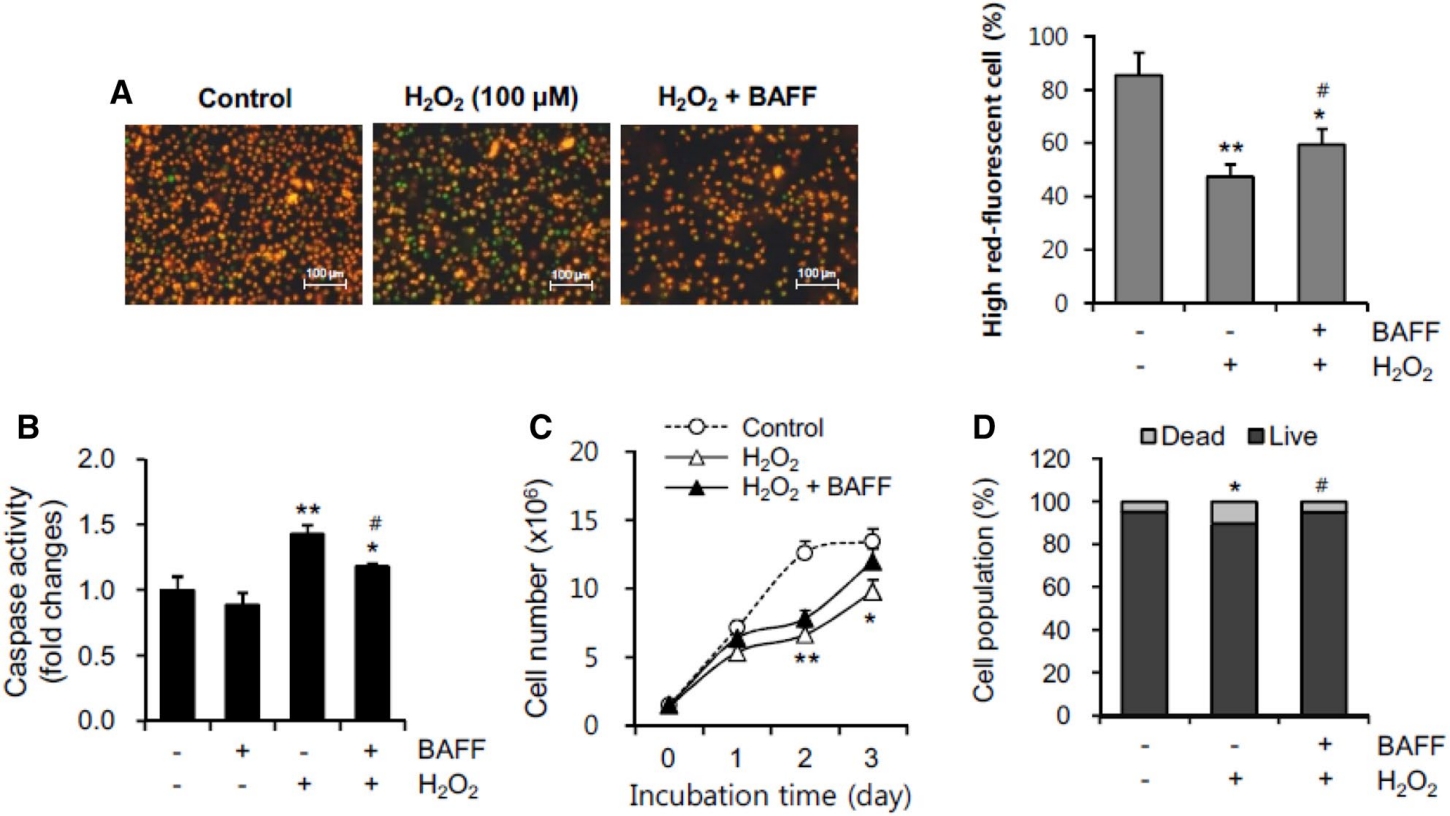
BAFF reduced MMP collapse and B cell death by the incubation with H_2_O_2_. (**A–D**) WiL2-NS cells were incubated in the RPMI 1640 medium with 100 µM H_2_O_2_ in the presence or absence of 20 ng/ml BAFF. Then, cells were stained with MitoProbe™ JC-1 reagent for the detection of MMP. Cells were observed and photographed with 400 × magnification under fluorescence microscope (**A**, left). Number of high-red fluorescent cells was counted (**A**, right). Cell lysates were prepared and caspase 3 activity was measured by using Ac-DEVD-pNA, caspase 3 substrate. Caspase 3 activity was normalized with protein concentration (**B**). Total number of cells (**C**) or dead cells (**D**) were counted with hemocytometer and estimated by trypan blue staining, respectively. All experiments were performed four times. Data in the bar (**A**, **B** and **D**) or line (**C**) graph represent the means ± SD. *p < 0.05; **p < 0.01; significant difference as compared to BAFF-untreated control with 10% FBS. ^#^*p* < 0.05; significant difference as compared to BAFF-untreated control with 1% FBS.

To confirm the inhibitory effect of BAFF on ROS-induced MMP collapse and cell death, WiL2-NS cells were incubated with 100 µM H_2_O_2_. MMP collapse by H_2_O_2_ treatment was assessed by staining cells with MitoProbe™ JC-1 reagent and by counting the number of live cells with high red fluorescence. H_2_O_2_ treatment decreased the MMP to about 46% as compared to 82% in the control group. When cells were incubated with 100 µM H_2_O_2_ in the presence of BAFF, MMP collapse was inhibited to about 60% (Fig. 4A). Caspase 3 assay results revealed that cell death in the presence of 100 µM H_2_O_2_ increased by about 1.5 times as compared to the control group. H_2_O_2_-induced caspase 3 activity was reduced about 1.2 times by treatment with BAFF (Fig. 4B). Incubation with H_2_O_2_ reduced the total cell number by about 40% compared to the control group, which was increased slightly in the presence of BAFF (Fig. 4C). Using trypan blue staining to assess the number of dead cells, the percentage of cell survival was reduced to about 89% as compared to about 96% in 10% FBS. When cells were incubated with H_2_O_2_ in the presence of BAFF, the percentage of cell survival was rescued to about 95% (Fig. 4D). This suggests that BAFF binding might activate or change molecules, or alter intracellular events to control H_2_O_2_-induced B cell death.

### Syk phosphorylation at Y525/526 was activated by BAFF or serum deprivation

**Figure 5:**
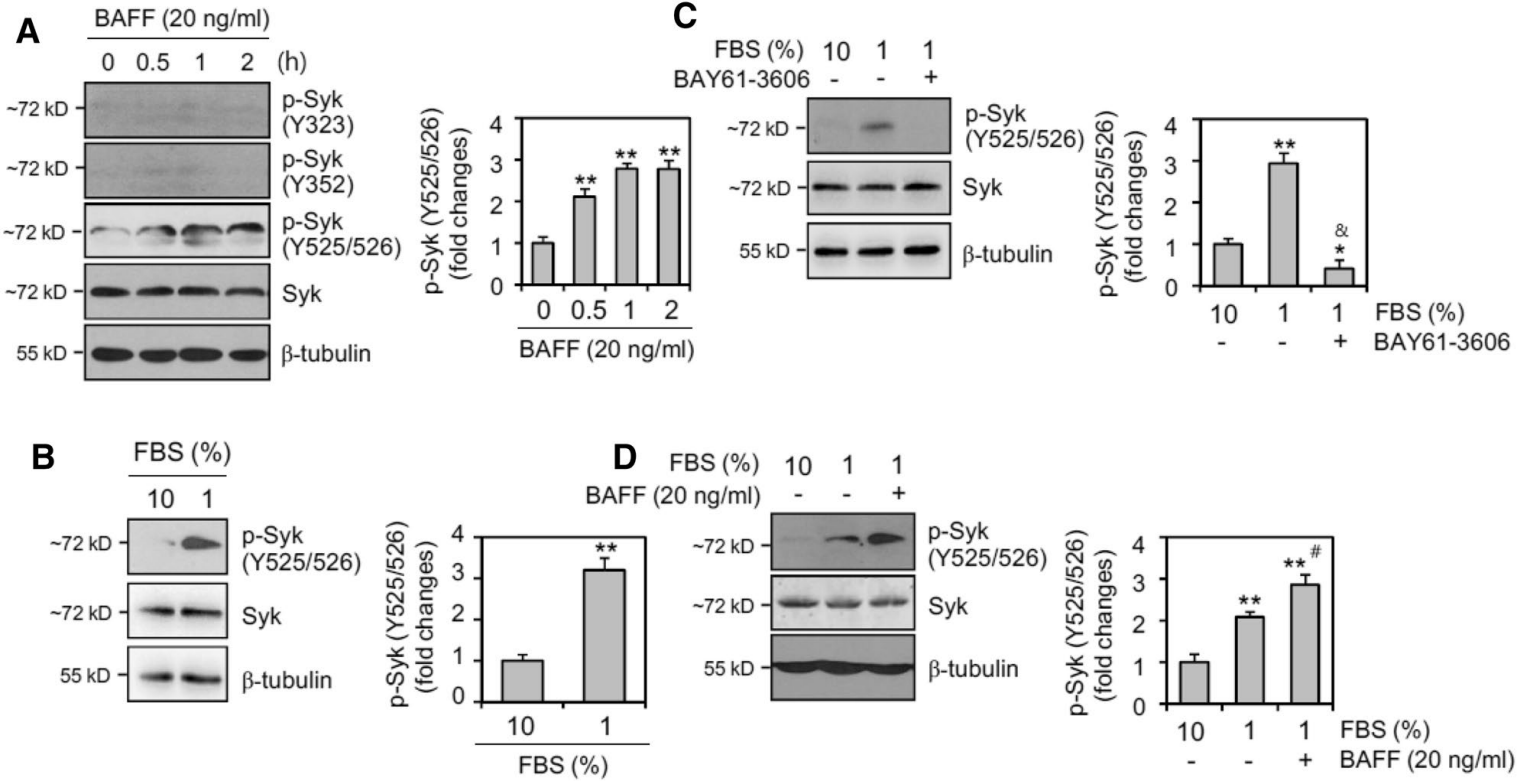
Spleen tyrosine kinase (Syk) is activated by the incubation with 1% FBS or BAFF. (**A**) WiL2-NS cells were incubated with 20 ng/ml BAFF for 0.5, 1 and 2 h. (**B–D**) WiL2-NS cells were incubated in the RPMI 1,640 medium with 10% or 1% FBS in the presence or absence of BAY61-3606, Syk inhibitor (**C**) or BAFF (**D**). Cell lysates were prepared and western blotting was used to detect each phosphorylated Syk at tyrosine (Y)323, Y352 and Y525/526. All experiments were performed four times. Processing (such as changing brightness and contrast) is applied equally to controls across the entire image. Each band for Syk phosphorylation at Y525/526 was quantified by using ImageJ 1.34. Data in the bar graph represent the means ± SD. *p < 0.05; **p < 0.01; significant difference as compared to BAFF-untreated control with 10% FBS. ^&^*p* < 0.05; significant difference as compared to BAY61-3606-untreated control with 1% FBS (**C**). ^#^*p* < 0.05; significant difference as compared to BAFF-untreated control with 1% FBS (**D**).

Because Syk can be activated by BAFF^42^ and plays an important role in B cell survival^42^, we sought to identify the tyrosine site of Syk that might be activated by BAFF binding to WiL2-NS lymphoblast B cells. When cells were incubated with 20 ng/ml of BAFF, we observed a time-dependent increase in Syk phosphorylation at Y525/526 (Fig. 5A). However, few changes were detected in Syk phosphorylation at Y323 or Y352. Syk phosphorylation at Y525/526 was enhanced by incubation with 1% FBS (Fig. 5B). When cells were incubated with 1% FBS in the presence of BAY61-3606, a Syk inhibitor, we observed a decrease in Syk phosphorylation at Y525/526 (Fig. 5C). In contrast, when cells were incubated with 1% FBS-containing medium in the presence of BAFF, Syk phosphorylation at Y525/526 was greatly increased compared to that in the control group with 1% FBS (Fig. 5D). These results indicate that BAFF provided a much stronger signal for B cell survival via Syk phosphorylation. Therefore, Syk activation might be required for B cells to survive in an environment that induces cell death.

### Syk inhibitor enhanced MMP collapse in serum-deprived conditions

**Figure 6:**
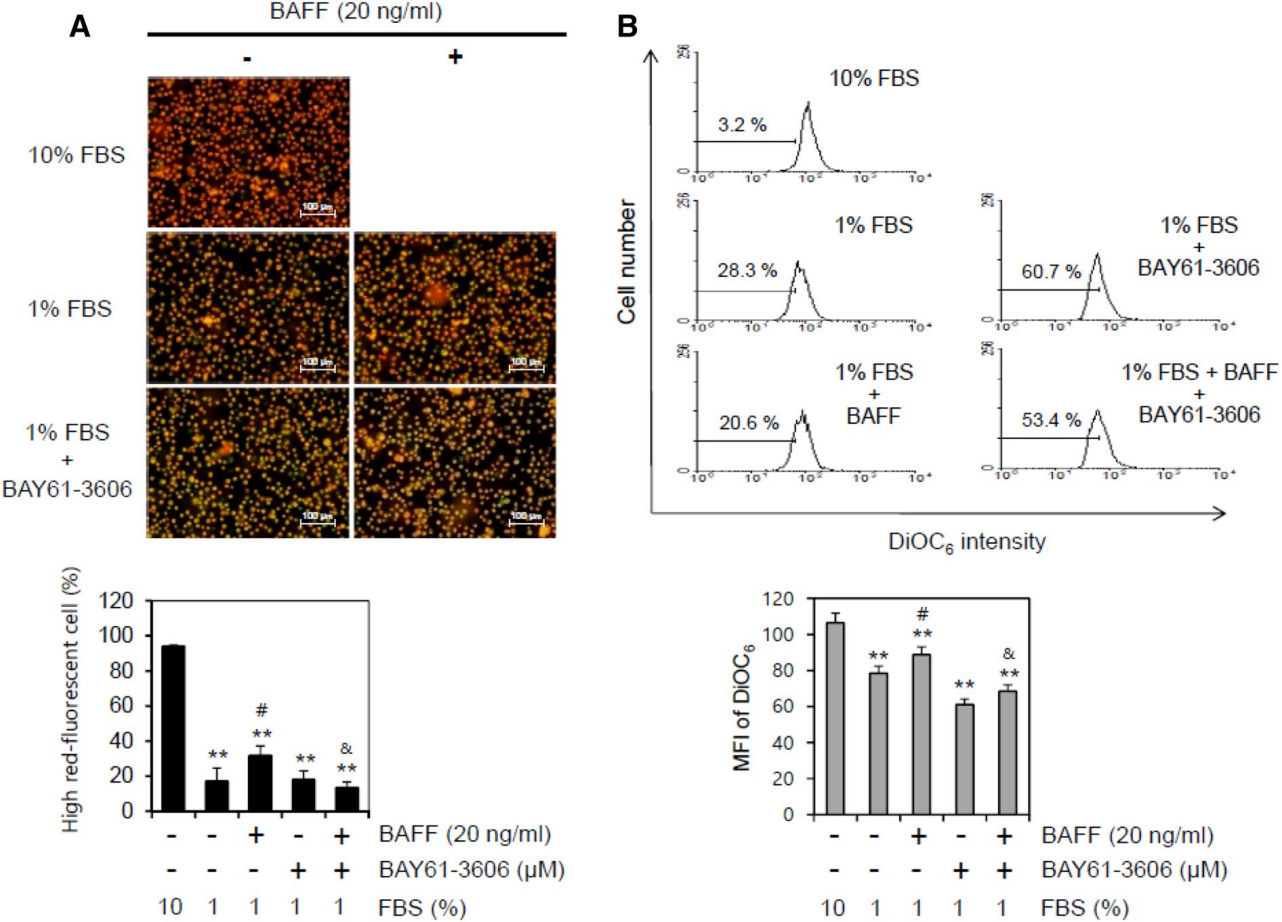
Syk inhibitor enhanced MMP collapse by the incubation with 1% FBS. (**A**, **B**) WiL2-NS cells were incubated in the RPMI 1640 medium with 10% or 1% FBS in the presence or absence of BAY61-3606, Syk inhibitor and/or 20 ng/ml BAFF. Then, cells were stained with MitoProbe™ JC-1 reagent (**A**) or DiOC_6_ (**B**) for the detection of MMP. Cells were observed and photographed with 400 × magnification under fluorescence microscope (**A**, top). Number of high-red fluorescent cells was counted and represented as bar graph with mean ± SD (**A**, bottom). Percentage of cells with low fluorescence of DiOC_6_ was analyzed by flow cytometry (**B,** top). Mean fluorescence intensity (MFI) in each histogram was analyzed with software FlowJo V10. MFI was represented as bar graph with the means ± SD (**B,** bottom). All experiments were performed four times. **p < 0.01; significant difference as compared to BAFF-untreated and BAY61-3,606-untreated control with 10% FBS. ^#^*p* < 0.05; significant difference as compared to BAFF-untreated and BAY61-3,606-untreated control with 1% FBS. ^&^*p* < 0.05; significant difference as compared to BAFF-treated and BAY61-3,606-untreated control with 1% FBS.

Because Syk is localized in the mitochondrial inter-membrane space where it can regulate respiratory activity^42^, we tested the role of BAFF-mediated Syk activation on MMP collapse in WiL2-NS cells using MitoProbe™ JC-1 reagent under serum-deprived conditions (1% FBS). Next, cells were incubated with 1% FBS-containing medium in the presence of BAY61-3606 and/or BAFF. The number of live cells with high red fluorescence was counted to assess the MMP (Fig. 6A, top). The results revealed that the MMP with 1% FBS decreased to about 18% as compared to 93% with 10% FBS. Treatment with BAFF significantly rescued the MMP to about 32%, which was inhibited by BAY61-3606. However, few changes were observed by treatment with BAY61-3606 alone (Fig. 6A, bottom). DiOC_6_ was also used to confirm the effect of the Syk inhibitor on MMP collapse. As shown in Fig. 6B, the fluorescence intensity of DiOC_6_ was decreased by incubation with 1% FBS. The percentage of cells with low fluorescence intensity of DiOC_6_ was increased to about 28.3%, which was reduced to about 20.6% by incubation with BAFF as compared to 3.2% with 10% FBS (Fig. 6B, top). The MFI of the cell population with 1% FBS was 78.5, which was increased to about 88.7 by incubation with BAFF as compared to about 106.6 with 10% FBS. Additionally, when cells were incubated with 1% FBS in the presence of BAY61-3606, the number of cells with low fluorescence intensity of DiOC_6_ significantly increased to about 60.7%, which was recovered to 53.4% by incubation with BAFF. The MFI of the cell population in the presence of BAY61-3606 was decreased to about 61.1, which was also enhanced to 68.5 by incubation with BAFF (Fig. 6B, bottom). This suggests that MMP collapse could be maintained by BAFF via Syk activation.

### BAFF-mediated inhibition of B cell death was attenuated by Syk inhibition

**Figure 7:**
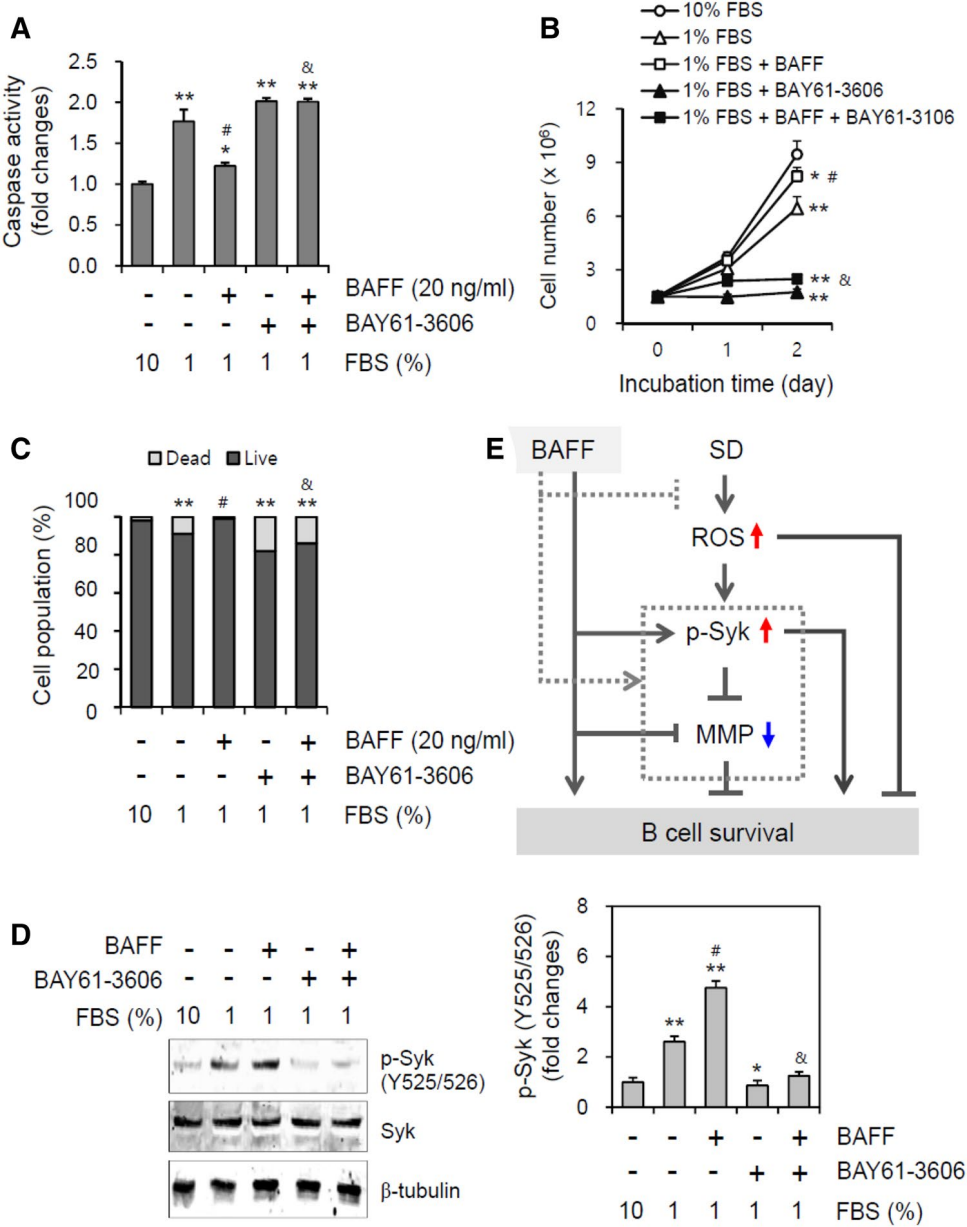
Syk inhibitor increased B cell death by the incubation with 1% FBS. (**A–D**) WiL2-NS cells were incubated in the RPMI 1,640 medium with 10% or 1% FBS in the presence or absence of BAY61-3,606, Syk inhibitor and/or 20 ng/ml BAFF. Cell lysates were prepared and caspase 3 activity was measured by using Ac-DEVD-pNA, caspase 3 substrate. Caspase 3 activity was normalized with protein concentration (**A**). Total number of cells (**B**) or dead cells (**C**) were counted with hemocytometer and estimated by trypan blue staining, respectively. Cell lysates were prepared and western blotting was used to detect phosphorylated Syk at tyrosine (Y) 525/526. Processing (such as changing brightness and contrast) is applied equally to controls across the entire image (**D**, left). Each band for Syk phosphorylation at Y525/526 was quantified by using ImageJ 1.34 (**D**, right). All experiments were performed four times. Data in the line (**B**) or bar (**A-D**) graphs represent the means ± SD. *p < 0.05; **p < 0.01; significant difference as compared to BAFF-untreated control with 10% FBS. ^#^*p* < 0.05; significant difference as compared to BAFF-untreated and BAY61-3,606-untreated control with 1% FBS. ^&^*p* < 0.05; significant difference as compared to BAFF-treated and BAY61-3606-untreated control with 1% FBS (**A**, bottom). (**E**) This is the scheme for the regulation of serum deprivation (SD)-associated B cell survival via Syk-dependent mitochondria membrane potential (MMP). Our findings are indicated by gray-dotted lines. Black lines are from the results reported already in the literature.

To confirm the role of Syk activation by BAFF in the inhibition of B cell death, WiL2-NS cells were incubated with 1% FBS-containing medium in the presence of BAY61-3606 and/or BAFF. Caspase 3 activity was enhanced by 1% FBS about 1.8 times as compared to that with 10% FBS. Caspase 3 activity induced by 1% FBS was reduced to about 1.2 times by treatment with BAFF, which was inhibited by BAY61-3606. Additionally, almost the same changes in caspase 3 activity were observed by treatment with BAY61-3606 alone (Fig. 7A). The total cell number was reduced to about 70% by incubation with 1% FBS as compared to that with 10% FBS. When cells were incubated with 1% FBS-containing medium in the presence or absence of BAFF, the total cell number decreased by incubation with 1% FBS was increased to about 90% by treatment with BAFF. However, the total cell number was highly reduced by treatment with BAY61-3606 in the presence or absence of BAFF (Fig. 7B). Trypan blue staining was used to determine the number of dead cells; the percentage of cell survival was calculated and was found to be reduced about 92% with 1% FBS as compared to about 98% with 10% FBS, which was inhibited by BAY61-3606. When cells were incubated with 1% FBS-containing medium in the presence of BAFF, the percentage of cell survival was rescued to about 99%, which was also inhibited by BAY61-3606 (Fig. 7C). These results were consistent with changes in Syk phosphorylation at Y525/526 (Fig. 7D, left). When cells were incubated with 1% FBS-containing medium, Syk phosphorylation at Y525/526 increased about 2.5-fold as compared to control with 10% FBS, which was inhibited by BAY61-3606. Syk phosphorylation at Y525/526 increased about 4.7-fold by incubation with 1% FBS in the presence of BAFF, which was also inhibited by BAY61-3606 (Fig. 7D, right). This suggests that BAFF might regulate B cell survival by controlling MMP via oxidative stress-induced Syk activation (Fig. 7E).

## Discussion

B cells play a role in the progression of autoimmune disease by releasing inflammatory cytokines, presenting autoantigens, producing antibodies, and ensuring interactions with T cells^43^. Therefore, many researchers are investigating how B cells can survive in the aggravation of autoimmune disease. ROS are increased in most inflammatory disease sites and are highly reactive due to their unpaired electron^11–14^. ROS that are excessively increased in inflammatory responses could affect cell fate by damaging various cellular components such as DNA, lipids, proteins^13,14^, and organelles^15^. For example, the electron transport chain in mitochondria can be disrupted in the middle of cell death^15^. BAFF enhances B cell survival, which is indispensable for B cell maturation and the enhancement of immune responses. Excessive responses by elevated BAFF levels lead to the accumulation of antigen–antibody immune complexes, which aggravate autoimmune diseases such as RA, SLE, and type 1 diabetes^1,9,10^. ROS can induce B cell activation, which is similar to the response mediated via B cell antigen receptors. Syk is a molecule that is activated by ROS^28,29^. However, little is known about the regulation of B cell survival by Syk activation-associated MMP control via ROS induction. BAFF mediates ROS control: here, we investigated whether B cell survival could be regulated via BAFF-mediated control of ROS and MMP collapse via Syk activation using a WiL2-NS human B cell line. Our results revealed that BAFF ameliorated SD-induced B cell death by the inhibition of MMP collapse through Syk activation. Therefore, Syk could be a key regulator for the maintenance of MMP by BAFF for human B cell survival.

BAFF can bind three receptors: BAFF-R, TACI, and BCMA. Among these, BAFF-R has binding affinity for only BAFF, and BCMA has the weakest binding affinity. TACI and BCMA also have affinity to APRIL, which has a similar structure to BAFF^1^. Our results revealed that BAFF binding was significant on the cell surface of WiL2-NS human B lymphocytes. While WiL2-NS cells expressed high levels of BAFF-R and low levels of TACI, no BCMA expression was detected. BAFF has a different binding affinity to each BAFF receptor, so it is necessary to determine how much each receptor contributes to BAFF-induced survival.

Although the exact cause of autoimmune disease is not well-understood, auto-antibodies from auto-reactive B cells that attack host cells are cited as one of the main causes^44,45^. To produce autoantibodies, B cells must overcome many harmful kinds of environments such as those with a high amount of ROS or MMP collapse, which threaten cell death in disease sites. BAFF enhances B cell survival, which is indispensable for B cell immune responses that aggravate autoimmune diseases ^1,9,10^. Our research, conducted in serum-deprived conditions, provides the groundwork for an experimental model to test the effect of therapeutic candidates on BAFF-mediated autoimmune disease-like states with ROS production and MMP collapse.

BAFF up-regulated MMP through AKT and PKC-β in mouse B cells^19^. BAFF also activated Syk^31^ in B cells of different species. BAFF expression was also increased by ROS in mouse macrophages^20^. Therefore, it is possible for BAFF expression to be regulated by Syk activation.

Syk belongs to the Syk family of tyrosine kinases and transmit signals from B-cell receptor (BCR), T-cell receptor (TCR), and BAFF receptors for various cellular responses^26^. Syk consists of many tyrosine residues that can potentially be phosphorylated. In human Syk, Y323, Y352, and Y525/526 are the most reported residues. Each of the tyrosine phosphorylation sites leads to a different downstream pathway. Phosphorylated Y323 becomes the binding site for the E3 ubiquitin ligase CBL, which mediates Syk ubiquitylation and degradation ^9,23,25^. Phosphorylated Y352 is the binding site for the PI3K regulatory subunit p85α N-terminal SH2 domain. It is also the binding site for the C-terminal SH2 domain of PLC-γ^24^. Y525/526 is located in the kinase domain, and its phosphorylation plays a crucial role in Syk activation^27^. Our results indicate that SD or BAFF treatment increased Syk phosphorylation at Y525/526. This suggests that BAFF-mediated cell survival could be dependent on Syk activation to control MMP collapse by SD.

Syk also contributes to B cell survival and differentiation^21,22^. Therefore, Syk activation in our study could be associated with different molecules to maintain cell survival. Syk might inhibit the activation of caspase-9 through AKT^13^. AKT plays an important role in B cell survival and is activated in a Syk-dependent pathway^46^. Syk also protects cells from apoptosis induced by oxidative or genotoxic stress by stabilizing the mRNA for Bcl-x(L), an antiapoptotic protein^32^. Syk also transduces BAFF survival signals via extracellular signal-regulated kinase (ERK) and PI3 kinase^31^.

At the same time, the possibility that Syk might activate cell death pathways cannot be ruled out. Syk-dependent PLCγ2 activation was required for acceleration towards apoptosis following oxidative stress^13^. Mule (also known as ARF-BP1, HUWE1, Ureb1, LASU1, and HECTH9) belongs to the E5-AP C terminus (HECT) domain-containing ubiquitin ligase (E3). Mule phosphorylation by Syk induces TNF-induced c-Jun N-terminal kinase (JNK) activation and cell death^47^. As such, it is necessary to define Syk-associated molecules by BAFF binding on cell surfaces in the inhibition of SD-induced MMP collapse.

In conclusion, our findings demonstrate that SD-induced ROS production causes MMP collapse through oxidative stress, which leads to cell death. Furthermore, this was attenuated by BAFF binding on BAFF receptors via Syk activation. Together, our results suggest that Syk activation by BAFF-BAFF receptor binding could regulate ROS production and inhibit MMP collapse and B cell death. These results provide additional evidence that BAFF or Syk may be valuable targets for B cell-mediated autoimmune disease therapies, such as the chemical KR33426^36^.

## Supplementary information

Supplementary Information.
