# Supplementary material for: BAFF attenuates oxidative stress-induced cell death by the regulation of mitochondria membrane potential via Syk activation in WiL2-NS B lymphoblasts
Source: Sci Rep. 2020 Jul 16;10:11784. doi: 10.1038/s41598-020-68628-5 (PMC7366908; doi:10.1038/s41598-020-68628-5)
Supplement: Supplementary file 1 — Supplementary Information. [file 41598_2020_68628_MOESM1_ESM.docx]

**BAFF attenuates oxidative stress-induced cell death by the regulation of mitochondria membrane potential via Syk activation in WiL2-NS B lymphoblasts**

Sojin Park^a^, Ju-Won Jang^a^ and Eun-Yi Moon^a,^ *

*^a^Department of Bioscience and Biotechnology, Sejong University, Seoul 05006, Republic of Korea*

Running title: BAFF attenuates B-cell death by regulating MMP via Syk activation

*Corresponding author

Eun-Yi Moon, Department of Bioscience and Biotechnology, Sejong University, 209 Neungdong-ro Kwangjin-Gu, Seoul 143-747, Republic of Korea.

Tel: +82 2 3408 3768; Fax: +82 2 466 8768.

E-mail address: [eunyimoon@sejong.ac.kr](mailto:eunyimoon@sejong.ac.kr) (E.Y. Moon)

**Supplementary information**

1. Corresponding author authorized that we checked western blots used in figure 5 for their compliance with the digital image and integrity policies (www.nature.com/srep/policies/index.html#digital-image).
2. Corresponding author authorized that we did not make the grouping of blots cropped from different parts of the same gel, or from different gels, fields, or exposures (e.g., using clear delineation either with dividing lines or white space).
3. Corresponding author authorized that we also did not make high-contrast (overexposure) of western blots. Blots are all from original blots by applying the process to change brightness and contrast equally across the entire image. For blots in figure 5, processing (such as changing brightness and contrast) is applied equally to controls across the entire image. We have mentioned this in figure legend of figure 5.
